# Identification of a 467 bp Promoter of Maize Phosphatidylinositol Synthase Gene (ZmPIS) Which Confers High-Level Gene Expression and Salinity or Osmotic Stress Inducibility in Transgenic Tobacco

**DOI:** 10.3389/fpls.2016.00042

**Published:** 2016-02-01

**Authors:** Hongli Zhang, Jiajia Hou, Pingping Jiang, Shoumei Qi, Changzheng Xu, Qiuxia He, Zhaohua Ding, Zhiwu Wang, Kewei Zhang, Kunpeng Li

**Affiliations:** ^1^Key Laboratory of Plant Cell Engineering and Germplasm Innovation, Ministry of Education, School of Life Science, Shandong UniversityJinan, China; ^2^Research Center of Bioenergy and Bioremediation, College of Resources and Environment, Southwest UniversityChongqing, China; ^3^Biology Institute of Shandong Academy of SciencesJinan, China; ^4^Maize Institute of Shandong Academy of Agricultural SciencesJinan, China

**Keywords:** abiotic stress, GUS analysis, inducible promoter, *Nicotiana benthamiana*, *Zea mays* L., *ZmPIS*

## Abstract

Salinity and drought often affect plant growth and crop yields. Cloning and identification of salinity and drought stress inducible promoters is of great significance for their use in the genetic improvement of crop resistance. Previous studies showed that phosphatidylinositol synthase is involved in plant salinity and drought stress responses but its promoter has not been characterized by far. In the study, the promoter (*pZmPIS*, 1834 bp upstream region of the translation initiation site) was isolated from maize genome. To functionally validate the promoter, eight 5′ deletion fragments of *pZmPIS* in different lengths were fused to GUS to produce *pZmPIS::GUS* constructs and transformed into tobacco, namely PZ1–PZ8. The transcription activity and expression pattern obviously changed when the promoter was truncated. Previous studies have demonstrated that NaCl and PEG treatments are usually used to simulate salinity and drought treatments. The results showed that PZ1–PZ7 can respond well upon NaCl and PEG treatments, while PZ8 not. PZ7 (467 bp) displayed the highest transcription activity in all tissues of transgenic tobacco amongst 5′ deleted promoter fragments, which corresponds to about 20 and 50% of *CaMV35S* under normal and NaCl or PEG treatment, respectively. This implied that PZ7 is the core region of *pZmPIS* which confers high-level gene expression and NaCl or PEG inducible nature. The 113 bp segment between PZ7 and PZ8 (-467 to -355 bp) was considered as the key sequence for *ZmPIS* responding to NaCl or PEG treatment. GUS transient assay in tobacco leaves showed that this segment was sufficient for the NaCl or PEG stress response. Bioinformatic analysis revealed that the 113 bp sequence may contain new elements that are crucial for *ZmPIS* response to NaCl or PEG stress. These results promote our understanding on transcriptional regulation mechanism of *ZmPIS* and the characterized PZ7 promoter fragment would be an ideal candidate for the overexpression of drought and salinity responsive gene to improve crop resistance.

## Introduction

Salinity and drought interferes plant growth as it causes osmotic stress and ion toxicity (Golldack et al., 2014). Global crop production is often affected by salinity or drought stress and the problem becomes increasingly serious (Munns and Tester, 2008; Tester and Langridge, 2010; Agarwal et al., 2013). Genetic improvement in crops by transgenic technology is considered as an effective way of solving this problem. An appropriate promoter that enables transgene expression at desired levels plays crucial roles in efficient production of transgenic plants (Potenza et al., 2004; Han et al., 2015; Tao et al., 2015). Isolation of various promoters and characterization of their expression patterns are necessary for transgenic breeding.

To date, promoter resources for crop genetic improvement are still quite limited. Constitutive promoters, such as *CaMV35S* promoter and maize ubiquitin promoter, are currently in use (Odell et al., 1985; Cornejo et al., 1993). *CaMV35S* promoter drives high-level gene expression in dicot plants, while maize ubiquitin promoter is more capable of driving gene expression in monocot plants. They are able to drive high-level transgene expression in almost all tissues and development stages in dicot or monocot plants, which results in not only excessive energy waste, but also morphological and physiological dysfunction frequently (Sinha et al., 1993; Cheon et al., 2004; Zhao et al., 2007). Inducible or tissue-specific promoters regulate target gene expression at specific conditions or tissues. Thus far some tissue-specific and stress-inducible promoters have been cloned and characterized. For example, *pF128* promoter drives high-level GUS expression specific in the seeds of transgenic foxtail millet and maize (Pan et al., 2015). The promoters of *rd29A* and *rd29B* genes in *Arabidopsis* respond to multiple stimuli including salinity and drought (Shinozaki and Shinozaki, 1993). *BADH* promoter from *Suaeda liaotungensis* exhibits salt induced activity (Zhang et al., 2008). *Rab16A* promoter could up-regulate GUS expression in transgenic rice under salt stress (Rai et al., 2009). *TsVP1* promoter from *Thellungiella halophila* displays strong activity in almost all tissues except the seeds and could be induced by salt stress in leaves and roots, especially root tips (Sun et al., 2010). *Dreb2* promoter responds to drought stress in wheat ancestors (Tavakol et al., 2014). Salt overly sensitive gene promoter *BjSOS2* from *Brassica juncea* responds to multiple stresses including salinity, drought and ABA (Kaur et al., 2015). However, the knowledge of inducible or tissue-specific promoters is still limited and the majority reported these specific promoters are weak ability of directing gene expression, which restricts their application.

Previous studies indicated that phosphoinositide (PI) signaling pathways are involved in environmental adaptation in higher plants, such as drought and salinity (Heilmann, 2009; Xue et al., 2009). Phosphatidylinositol (PtdIns) synthesis is the starting point of PI signaling pathway, which is synthesized by phosphatidylinositol synthase (PIS) from cytidinediphospho-diacylglycerol and D-myo-inositol (Löfke et al., 2008; Heilmann, 2009). The maize phosphatidylinositol synthase gene (*ZmPIS*, GRMZM2G110646, MaizeGDB) has 11 exons and 10 introns. The cDNA sequence is 1707 bp including 648 bp protein coding sequence, 313 bp 5′ UTR and 746 bp 3′ UTR. Based on the length of its 5′ UTR, we speculated the transcription start site of *ZmPIS* may be located in the translation initiation codon ATG upstream of approximately 313 bp. Previously, our laboratory isolated an 1164 bp *ZmPIS* cDNA sequence containing complete protein coding region (GenBank accession no. AY370763). The transcript abundance of *ZmPIS* is obviously increased by PEG, coldness and phytohormones (Sui et al., 2008). Overexpression of *ZmPIS* in tobacco and maize improves drought stress tolerance through altering membrane lipid composition and increasing ABA synthesis (Zhai et al., 2012; Liu et al., 2013). However, the promoter region of this gene has not been characterized by far. Isolation and characterization of *ZmPIS* promoter will provide novel insight into understanding of PI signaling pathway and promoter resources for crop genetic improvement.

In this study, expression patterns and transcriptional activities of 5′ deleted *ZmPIS* promoter fragments in different sizes were characterized in transgenic tobacco. A 467 bp fragment with high promoter activity that enables salt or osmotic stress inducible gene expression and a novel 113 bp *cis*-regulatory core region that is critical for salt or osmotic stress response were identified.

## Materials and Methods

### Isolation of *ZmPIS* Promoter Sequence

Primers were screened from the 2 kb 5′ flanking region of the ATG start codon of *ZmPIS* (GenBank accession. no. AY370763). The sequence was amplified from maize genomic DNA with primers pZmPISFR (**Table 1**) and confirmed by sequencing. Finally, the 1834 bp fragment upstream of the translation initiation site (ATG) of *ZmPIS* was obtained by polymerase chain reaction (PCR) amplification using PZ1 primers (**Table 1**) and considered as the full-length promoter.

**Table 1:** Polymerase chain reaction (PCR) primers used in the present study.

Name	Forward (5′–3′)	Reverse (5′–3′)
pZmPISFR	gcccgctatgagcctaaac	tccagaggcgtaccgataa
PZ1	cgcggatccgcccgctatgagcctaaa	ccg*gaattc*tttgccagagggcaattg
PZ2	taaggatccgttctacttcttgaagg	ccg*gaattc*tttgccagagggcaattg
PZ3	aaaggatccccactagggcaatgggaa	ccg*gaattc*tttgccagagggcaattg
PZ4	acgggatccggcaatagaatgaaaaa	ccg*gaattc*tttgccagagggcaattg
PZ5	ctaggatccgcgcaaccgaacacgccg	ccg*gaattc*tttgccagagggcaattg
PZ6	aatggatcctatttggctatctgtat	ccg*gaattc*tttgccagagggcaattg
PZ7	ccgggatccatgatgcaaaaactagg	ccg*gaattc*tttgccagagggcaattg
PZ8	aagggatccttgccacaattcagat	ccg*gaattc*tttgccagagggcaattg
p35SFR	aatggatccaagtctcaatagcccttt	tgagaattccgtattggctagagcagc
p113FR	cgcggatccatgatgcaaaaactag	aaaactgcagcaaataaagcttgaacta
HPTFR	cgtctgctgctccatacaa	tgtcctgcgggtaaatagc

*The underlined sites are the sites for the digestion of restriction enzymes BamH1. The underlined italicized sites are the sites for the digestion of restriction enzymes EcoR1*.

### Bioinformatic Analysis of the Promoter Sequence

**FIGURE 1:**
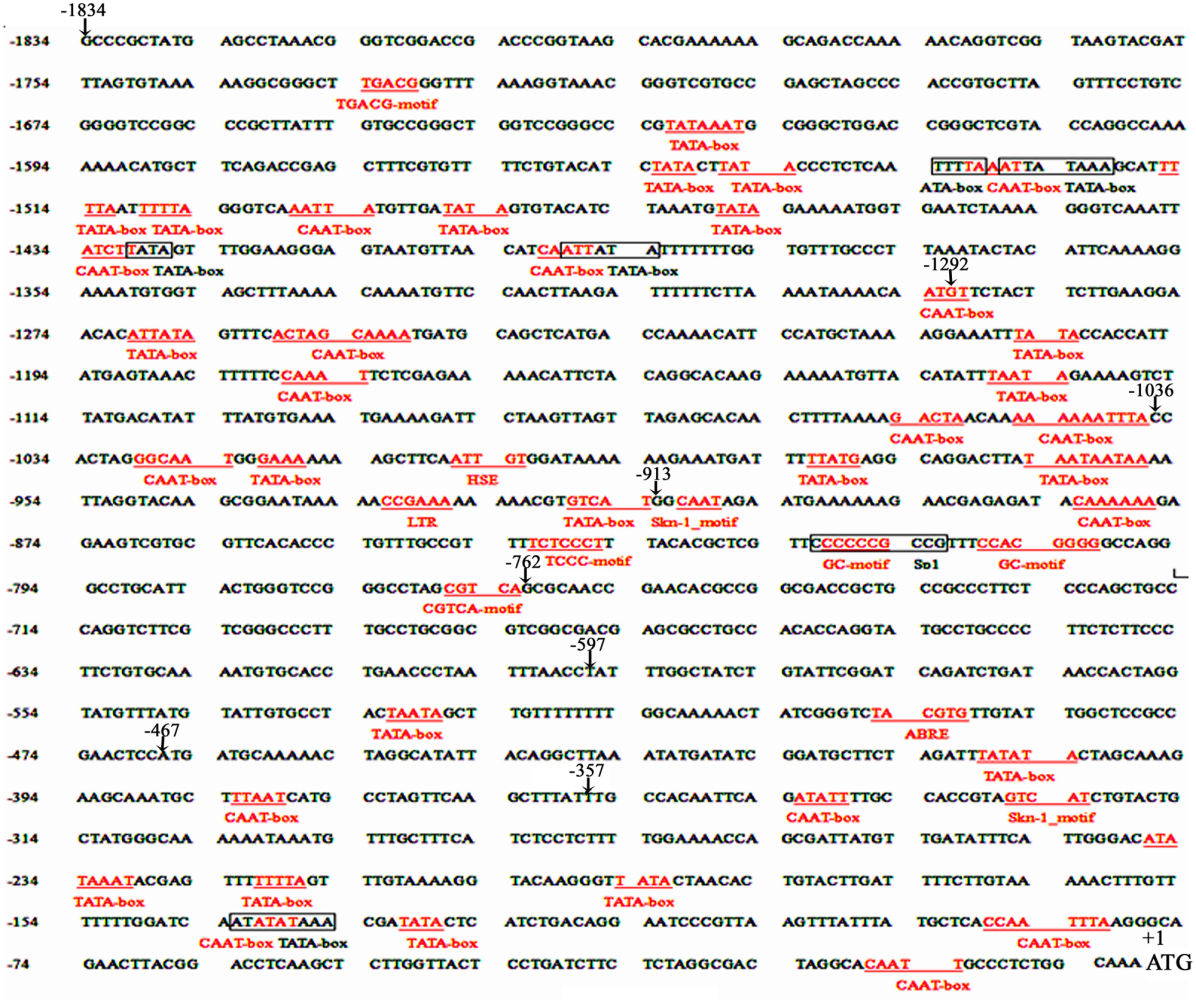
**Nucleotide sequence of the *pZmPIS***. The A of translation initiation site (ATG) of *ZmPIS* is numbered as +1. Known *cis*-acting elements are shown in underline or border. The descriptions of elements see **Table 2**. An arrow above the sequence indicates the start point of different deletion fragments.

The *cis*-acting elements of 1834 bp *ZmPIS* promoter (*pZmPIS*) sequence were analyzed using online programs PLACE (^1^Higo et al., 1999) and PlantCARE (^2^Lescot et al., 2002). The position and description of *cis*-acting elements are present in **Figure 1** and **Table 2**.

**Table 2:** Known *cis*-acting elements in the *pZmPIS* using the PlantCARE and PLACE databases.

*Cis*-elements	Description	No
CAAT-box	Common *cis*-acting element in promoter and enhancer regions	16
CTAG-motif	Unknown	1
HSE	*Cis*-acting element involved in heat stress responsiveness	1
LTR	*Cis*-acting element involved in low-temperature responsiveness	1
Skn-1_motif	*Cis*-acting regulatory element required for endosperm expression	2
TATA-box	Core promoter element around -30 of transcription start	25
TCCC-motif	Part of a light responsive element	1
TGACG-motif	*Cis*-acting regulatory element involved in the MeJA-responsiveness	1
ABRE	*Cis*-acting element involved in the abscisic acid responsiveness	1
CGTCA-motif	*Cis*-acting regulatory element involved in the MeJA-responsiveness	1
GC-motif	Enhancer-like element involved in anoxic specific inducibility	2
Sp1	Light responsive element	1

*The position of *cis*-acting elements in the sequence of *pZmPIS* was shown in **Figure 1**. MeJA, methyl jasmonate; Sp1, Sp1 transcription factor family binding sites*.

### Construction of Promoter*::GUS* Fusion Plasmids

**FIGURE 2:**
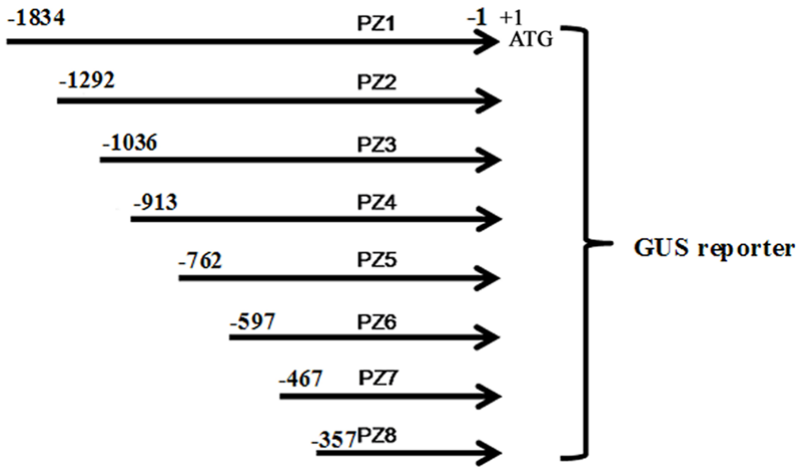
**The constructs of the truncated fragments of *pZmPIS* fused with *GUS***. A series of 5′ deleted fragments of *pZmPIS* were placed upstream of the GUS reporter gene. The numbers indicate the nucleotide position from the translational initiate site, ATG (A as +1).

Eight 5′ deleted fragments (PZ1–PZ8) in different size (-1834, -1292, -1036, -913, -762, -597, -467, and -357 bp to -1 bp; the translation initiation site was designated as “+1”; **Figure 2**) were amplified by PCR from the 1834 bp promoter region of *ZmPIS* with the oligonucleotide primers listed in **Table 1**. For the generation of *pZmPIS::GUS* constructs, the PCR products were subsequently constructed into the pCAMBIA1391Z vector (Cambia, Australia) with *BamH1*/*EcoR1* restriction sites and confirmed by sequencing. The resulting constructs were used for tobacco (*Nicotiana benthamiana*) stable transformation.

The minimal *CaMV35S* promoter fragment (-46 ∼ +10 bp) was amplified by PCR using primers p35SFR (**Table 1**). The amplified fragment was confirmed by sequencing and then the fragment was cloned into the *Pst1*/*Spe1* sites upstream of the *GUSA* reporter gene in vector pCAMBIA1304 (Cambia, Australia). The plasmid was designated as P-mini 35S. The 113 bp sequence of the *ZmPIS* promoter (-467 ∼-355 bp) was cloned by PCR using the primers p113FR (**Table 1**) and then confirmed by sequencing. The amplified fragment was inserted into the *BamH1*/*Pst1* sites of the P-mini 35S vector. The construct was named P-113 bp mini 35S. These plasmids were used for tobacco transient assay.

In addition to these constructs above, pCAMBIA1304 vector containing the *CaMV35S* promoter upstream from the *GUSA* reporter gene was used as a positive control.

### Culture and Stable Transformation of Tobacco

Tobacco seeds were sterilized with 70% ethanol for 1 min, 10% NaClO for 10 min, then washed with sterile water for 5–6 times, 5–7 min every time. The seeds were germinated at 25°C for 7 days. Then 7-days-old seedlings were transferred into culture bottles containing nutrient medium (KNO_3_ 9.4 mM, NH_4_NO_3_ 10.3 mM, MgSO_4_ 0.75 mM, KH_2_PO_4_ 0.62 mM, CaCl_2_ 1.5 mM, KI 2.59 μM, H_3_BO_3_ 65 μM, MnSO_4_ 50 μM, Na_2_MoO_4_ 0.62 μM, ZnSO_4_ 17.39 μM, CuSO_4_ 0.05 μM, CoCl_2_ 0.052 μM, Fe-EDTA 50 μM, inositol 0.27 mM, nicotinic acid 2.03 μM, VB_6_ 1.22 μM, VB_1_ 0.15 μM, Gly 13 μM, sucrose 30 g/L, agar 0.7%, pH 6.0) and grown in a growth chamber at 25°C with a cycle of 16 h light (220–260 μmol m^-2^ s^-1^) for 6 weeks until transformation.

The pCAMBIA1304 and recombinant plasmids PZ1–PZ8 were introduced into the *Agrobacterium tumefaciens* strain GV3101 using a freeze-thaw method. The *A. tumefaciens*-mediated transformation of tobacco leaf disks was performed as previously described by Voelker et al. (1987) with minor modifications. Transformed shoots were screened on MS medium supplemented with 1.0 mg/L 6-benzylaminopurine, 0.1 mg/L indole-3-acetic acid, 400 mg/L cefotaxime, and 15 mg/L hygromycin B. Regenerated shoots were rooted on MS medium containing 200 mg/L cefotaxime and 15 mg/L hygromycin B. The regenerated plants were cultivated in soil under 25–28°C (day) and 19–22°C (night) with a cycle of 16 h of light (220–260 μmol m^-2^ s^-1^). The T0 positive transformants were screened out for propagation by PCR of the hygromycin resistant gene harbored in the pCAMBIA1391Z and pCAMBIA1304 vectors with the primers HPTFR (**Table 1**) and GUS staining (Supplementary Figure S1). The homozygous transgenic lines of T3 generation with a single copy of promoter*::GUS* insert were selected for subsequent analyses via segregation ratio analysis.

### Salinity and Osmotic Stress Treatments

Transgenic tobacco plants were grown in a bottle containing 1 L 1/2 MS liquid medium (pH 6.0) under 25–28°C (day) and 19–22°C (night) with a cycle of 16 h light (220–260 μmol m^-2^ s^-1^). Sixty-days-old tobacco plants were subjected to stress treatments. For salinity, whole plants were incubated in the liquid 1/2 MS medium supplemented with 200 mM NaCl for 1, 3, 6, 12, 24, 48, and 72 h. For osmotic stress, tobacco plants were incubated in the liquid 1/2 MS medium supplemented with 18% (w/v) polyethylene glycol 6000 (PEG 6000). The control plants were grown in 1/2 MS liquid medium. Leaf tissues were sampled for GUS histochemical staining immediately and frozen in liquid nitrogen and stored at -80°C for GUS fluorometric assays. All the experiments were repeated three times with independent samples.

### Histochemical and Fluorometric GUS Assays

Three independent transgenic lines and at least five individual plants from each construct were used for *GUS* expression assay. Histochemical GUS staining was performed as described by Jefferson et al. (1987) with minor modifications. Briefly, the tissues were incubated in staining solution containing 50 mM sodium phosphate buffer (pH 7.0), 0.5 mM potassium ferrocyanide, 0.5 mM potassium ferricyanide, 0.1% Triton X-100, 10 mM EDTA, and 1 mM 5-bromo-4-chloro-3-indolyl-β-D-glucuronic acid (X-Gluc; Sangon, Shanghai, China). Following vacuum infiltration, the samples were incubated at 37°C for 24 h. The tissues decolorized in 70% ethanol were observed and recorded using a digital still camera (Sony, DSC-F828). Fluorometric assay of GUS activity was performed as described by Jefferson et al. (1987) with minor modifications. In brief, leaf tissues were homogenized in a 4°C extraction buffer containing 50 mM sodium phosphate (pH 7.0), 0.1% Triton X-100, 10 mM EDTA, 0.1% sodium lauryl sarcosine and 10 mM dithiothreitol. After centrifugation at 10000 *g* for 15 min at 4°C the activity of the supernatant was detected in working buffer containing 1 mM 4-methylumbelliferyl-b-glucuronide (4-MUG, Sigma, USA) at 37°C. The reaction was terminated by adding 200 mM Na_2_CO_3_ to a final concentration of 180 mM. The fluorescence was quantified using a fluorescence spectrophotometer (HITACHI F-4600, Japan) at the excitation and emission wavelengths of 365 and 455 nm, respectively. Protein concentration was determined as described by Bradford (1976). The GUS activity was normalized with five 4-MU standards (10 mM, 1 mM, 100 nM, 50 nM, and 10 nM) under control conditions and calculated as nmol of 4-MU per mg protein per minute.

### GUS Transient Assay in Tobacco Leaves

GUS transient assay was performed using the leaves of 7-weeks-old tobacco plants as described by Yang et al. (2000). *A. tumefaciens* GV3101 of each construct was grown on YEP medium containing rifampicin (50 mg/L) and kanamycin (50 mg/L) at 28°C for 18 h. The bacteria were harvested by centrifugation for 15 min at 6000 *g* and re-suspended in the transformation buffer (10 mm MES, pH 5.6, 10 mm MgCl_2_, 100 μm acetosyringone) to an OD 600 of 0.6 for leaf infiltration. After agro-infiltration at the abaxial surfaces of tobacco leaves using a needleless syringe, the plants were maintained in a moist chamber at 25°C for 2 days. For salinity and osmotic stress, the infiltrated leaves were incubated in the liquid 1/2 MS medium supplemented with 200 mM NaCl and 18% (w/v) PEG 6000 for 24 h, respectively. The infiltrated leaves grown in the liquid 1/2 MS medium were treated as control. Finally, at least 15 independent infiltrated leaves from different plants were used for histochemical GUS staining and fluorometric assay of GUS activity. The entire experiments were repeated three times.

### Data Analysis

All GUS fluorometric assays were repeated at least three times, and the results were expressed as mean values ± SD (standard deviation). The statistical significance of quantitative data was determined using Student’s *t*-test (*n* = 3, *P* < 0.05; Sigmaplot 12.0) at a 95% confidence level.

## Results

### Isolation and Sequence Analysis of the *pZmPIS*

Based on the annotation of maize genome, the 1834 bp promoter sequence of *ZmPIS* upstream of the translation initiation site (ATG) was isolated from maize genomic DNA. We analyzed the promoter sequence for the presence of putative *cis*-acting elements through PLANTCARE and PLACE databases. The results showed that the 1834 bp promoter sequence contains a number of TATA-box and CAAT-box core *cis*-acting elements. Some *cis*-acting elements in response to multiple environmental stimuli were also present, including two types of light responsive elements [TCCC-motif and Sp1 (Sp1 transcription factor family binding sites)], two types of MeJA (methyl jasmonate) responsive elements (TGACG-motif and CGTCA-motif), two known elements required for endosperm expression (Skn-1_motif), a enhancer-like element involved in anoxic specific inducibility (GC-motif), a heat stress responsive element (HSE), a low-temperature responsive element (LTR), an abscisic acid responsive element (ABRE) and a unknown element CTAG-motif (**Figure 1** and **Table 2**). However, known salinity or osmotic stress inducible *cis*-acting elements were not harbored in the *pZmPIS* sequence, although the expression of *ZmPIS* could be obviously induced by NaCl or PEG treatment (Sui et al., 2008; Zhai et al., 2012; Liu et al., 2013).

### Activity of 5′ Deleted Fragments of *ZmPIS* Promoter in Transgenic Tobacco

Six to ten independent transgenic lines for each construct (PZ1–PZ8 and *CaMV35S*) were screened in T2 generation by histochemical GUS staining. Three transgenic lines segregating 3:1 for each construct were selected, and the T3 homozygous generation was chosen for further analysis. The results of GUS staining showed that PZ1–PZ8 promoter fragments were capable of directing GUS expression, but their promoter activities in transgenic tobaccos were obviously different.

**FIGURE 3:**
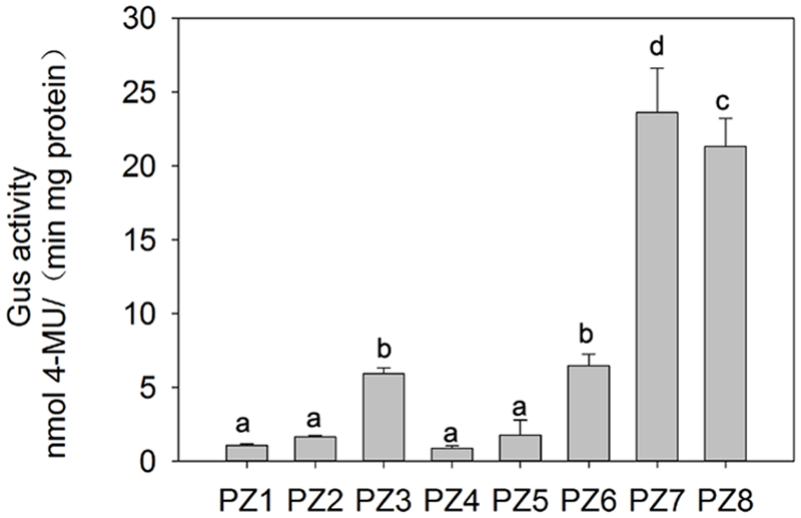
**Fluorometric GUS assays of PZ1–PZ8 transgenic tobacco plants**. The values are the mean ± standard deviation from three independent transgenic lines and each line five individual plants for each construct. Different lowercase letters above the bars indicate significant differences at *P* < 0.05.

In order to evaluate the transcriptional activities of PZ1–PZ8 under normal condition and identify core functional region of *ZmPIS* promoter, fluorometric GUS assays were further performed with transgenic tobacco leaves from different constructs (**Figure 3**). The promoter activities of PZ1–PZ6 were relatively weak, while PZ7 and PZ8 were able to drive strong GUS expression. Among them, the GUS expression driven by PZ7 was obviously higher than these of PZ1–PZ6 and PZ8, which corresponds to approximately 20 folds of GUS activity of full length promoter PZ1 and about 4 and 1.1 folds of PZ6 and PZ8. These results suggested that the 131 bp (-467 ∼-597 bp) sequence between PZ6 and PZ7 may contain *cis*-acting elements that inhibit gene expression, and PZ7 fragment (467 bp, -467 ∼-1 bp) may be the core functional region of *ZmPIS* promoter which contains multiple core *cis*-acting elements CAAT-box and TATA-box (**Figure 1**).

### Expression Pattern of PZ1 and PZ7 Transgenic Tobacco

**FIGURE 4:**
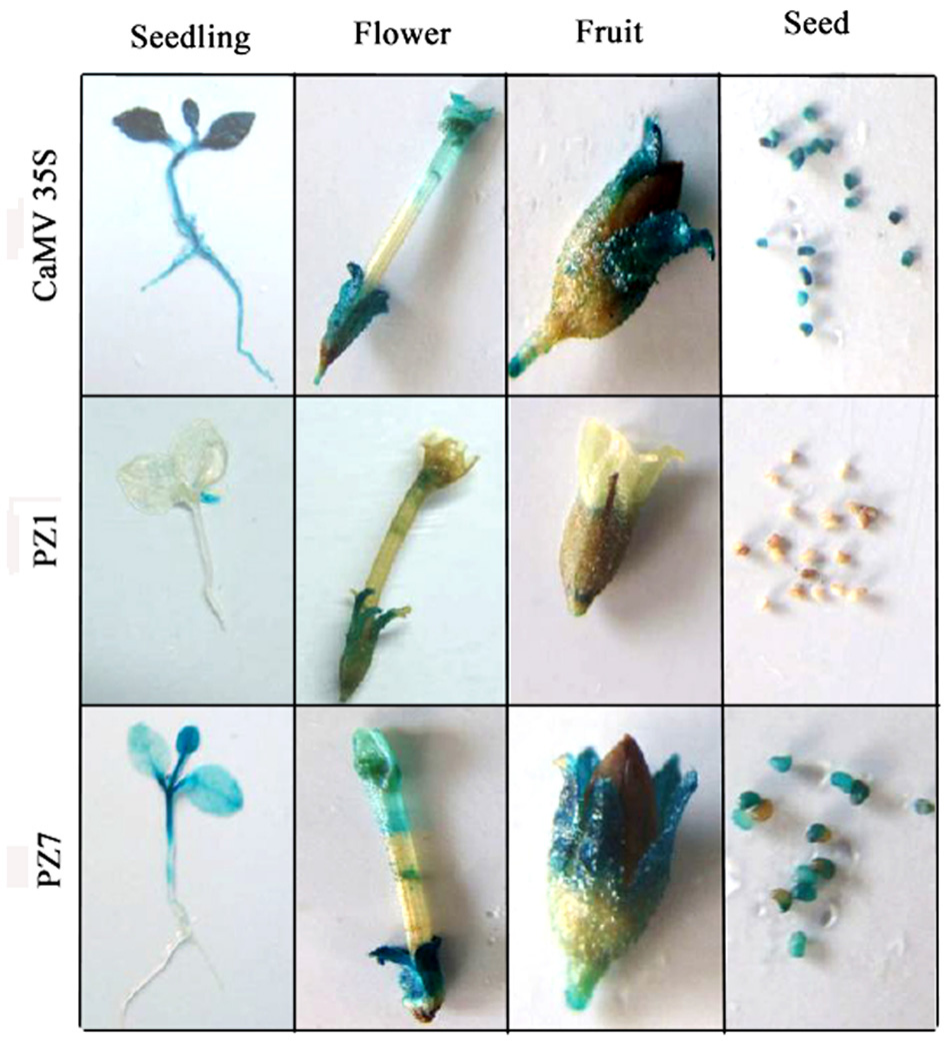
**Histochemical GUS staining in tissues of PZ1, PZ7, and *CaMV35S* transgenic tobacco**. Two-weeks-old tobacco seedlings, flowers, fruits, and seeds were incubated in staining solution at 37°C for 24 h. Then the samples were observed and photographed after decolorization.

To profile the expression of transgenic tobacco plants driven by PZ1 (full length promoter), PZ7 and *CaMV35S* under normal condition, the 2-weeks-old independent transgenic tobacco plants, flowers, fruits, and seeds were subjected to histochemical staining (**Figure 4**). *CaMV35S* transgenic plants displayed the highest GUS expression intensity among the tested constructs. GUS expression activity for PZ1 was the weakest in all tissues and almost not detected in the roots, stems and cotyledons of 2-weeks-old seedlings and seeds. The deletion of 1367 bp (-1834 to -467 bp) resulted in significantly strong GUS activity in all tissues. High GUS expression intensity was detected in almost all tissues of PZ7 plants including the leaves, cotyledon and stem of 2-weeks-old seedlings, and petals, sepals, stigma, and seed except the roots of 2-weeks-old seedlings. Taken together, the promoter deletion segment PZ7 was sufficient to drive high-level gene expression in transgenic tobacco under normal condition.

### Salinity and Osmotic Stress Responses Mediated by 5′ Deleted Fragments of *pZmPIS* in Transgenic Tobacco

**FIGURE 5:**
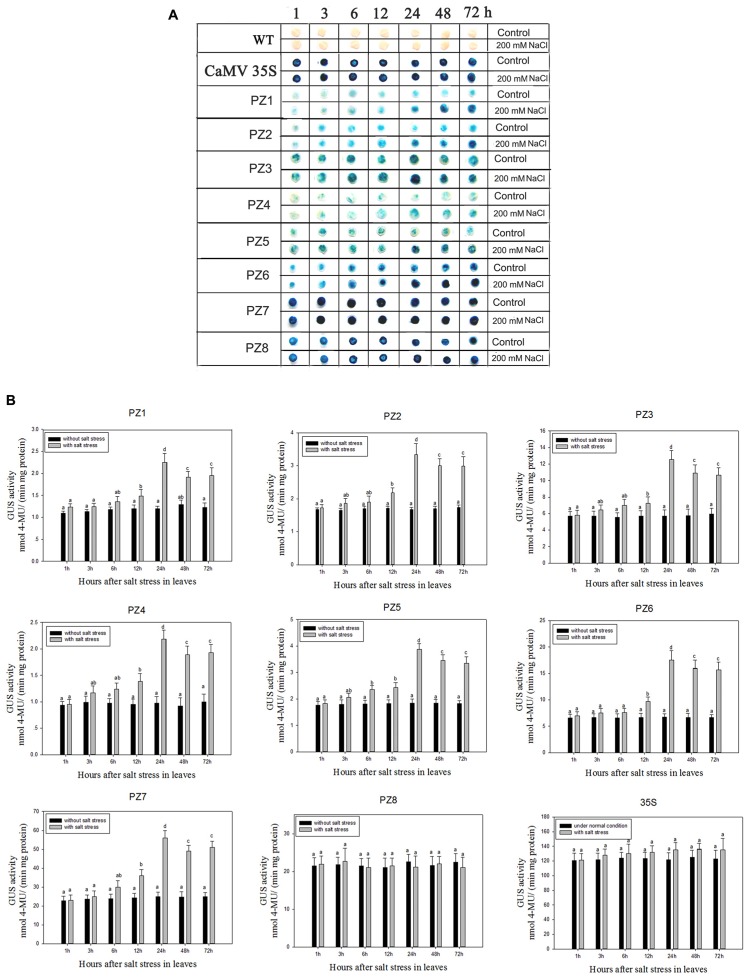
**GUS histochemical staining and fluorescent quantitative analysis of transgenic tobacco expressing eight truncated promoter of *pZmPIS::GUS* under normal and salt stress conditions. (A)** GUS histochemical staining; **(B)** GUS fluorescent quantitative analysis. The tobacco plants were incubated in the liquid 1/2 MS medium supplemented with 200 mM NaCl for 1, 3, 6, 12, 24, 48, and 72 h. The plants grown in the liquid 1/2 MS medium were treated as control. Values represent the mean ± standard deviation from three independent transgenic lines and each line five individual plants for each construct. Different lowercase letters above the bars indicate significant differences at *P* < 0.05.

**FIGURE 6:**
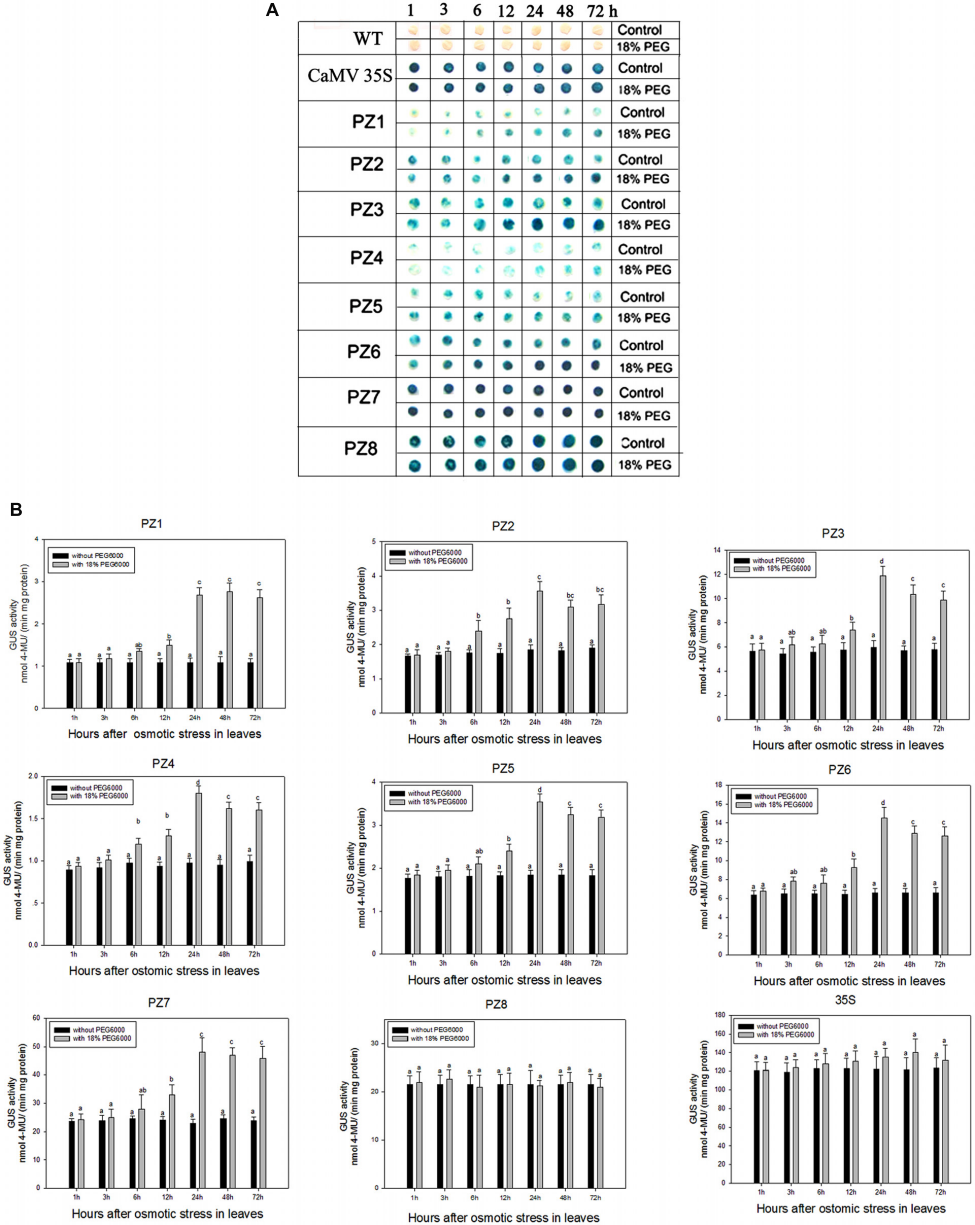
**GUS histochemical staining and fluorescent quantitative analysis of transgenic tobacco expressing eight truncated promoter of *pZmPIS::GUS* under normal and osmotic stress conditions. (A)** GUS histochemical staining. **(B)** GUS fluorescent quantitative analysis. The tobacco plants were incubated in the liquid 1/2 MS medium supplemented with 18% (w/v) PEG 6000 for 1, 3, 6, 12, 24, 48, and 72 h. The plants grown in the liquid 1/2 MS medium were treated as control. Values represent the mean ± standard deviation from three independent transgenic lines and each line five individual plants for each construct. Different lowercase letters above the bars indicate significant differences at *P* < 0.05.

The transcript abundance of *ZmPIS* can be regulated by multiple abiotic stresses including drought and salt (Sui et al., 2008; Zhai et al., 2012; Liu et al., 2013). However, bioinformatic analysis indicated that the 1834 bp *pZmPIS* seguence does not harbor any known salinity or osmotic stress inducible *cis*-acting elements. In order to investigate the regulatory machinery involved in *ZmPIS* activation upon salinity or osmotic stresses, promoter activities of PZ1–PZ8 were determined in leaves of 60-days-old transgenic tobacco plants subject to 200 mM NaCl or 18% PEG 6000 treatment in a time-course experiment, respectively. Positive (transformed *CaMV35S* promoter tobacco) and negative (wild type) control seedlings were also treated in parallel. Because of the original high GUS expression levels in leaves of 35S, PZ7 and PZ8 transgenic tobacco plants, it is difficult to see an obvious change in expression level just by GUS staining (**Figures 5A** and **Figures 6A**). In order to make clear the difference of GUS expression between the series of mutant before and after stresses, we also measured GUS enzyme activity (**Figures 5B** and **Figures 6B**). The results showed that there was no obvious difference between stress-treated groups and control groups after 1, 3, and 6 h stress treatments. PZ1–PZ7 plants started a stress-induced tendency after 12 h treatment, and the GUS expression intensity of PZ1–PZ7 plants displayed a significant increase compared to the controls after 24, 48, and 72 h salinity or osmotic stress treatments. GUS enzyme activities of PZ1–PZ7 were obviously induced up to more than twofolds in the leaves under salinity or osmotic stress treatment for 24 h and maintained at a relatively high expression level with 48 and 72 h treatments. However, GUS staining intensity and fluorometric activity of the leaves in PZ8 and *CaMV35S* transgenic tobacco were stable during the stress treatment. Therefore, PZ1–PZ7 displayed obviously salinity and osmotic stress-inducing activity, while PZ8 did not. These results suggested that the 110 bp fragment of *pZmPIS* between PZ7 and PZ8 (-467 to -357 bp) may contain functional elements involved in salinity or osmotic stress response. Therefore, *ZmPIS* promoter could well respond to salinity or osmotic stress. Notably, among transgenic tobacco lines of 5′ deleted *pZmPIS* fragments, PZ7 had the highest promoter activity and reached 20 times more than PZ1 and 3 times more than PZ6 under both normal and salinity or osmotic stress conditions. Under normal condition, the activity of PZ7 was about 1.1 fold of PZ8 and 0.2 fold of *CaMV35S*, while reached twofold more than PZ8 and about 50% of *CaMV35S* promoter under salinity or osmotic stress. This implied that PZ7 (467 bp; -467 to -1 bp) enables high level gene expression and salinity or osmotic stress inducibility.

### The 113 bp *cis*-Regulatory Region was Sufficient for the Salinity and Osmotic Stress Responses

**FIGURE 7:**
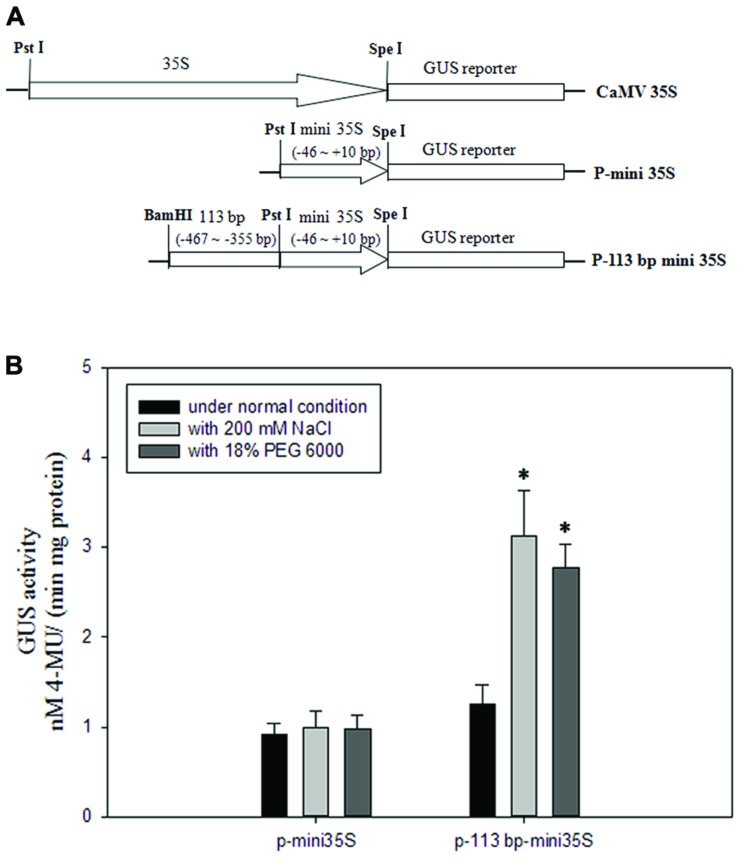
**GUS transient assays in tobacco leaves. (A)** Fusion constructs used in the transient assay. The *CaMV35S* represent 35S full length promoter. P-mini 35S represent the mini 35S (-46 to +10 bp) promoter. The test construct P-113 bp mini 35S in which the 113 bp region (-467 to -355 bp) identified in the *pZmPIS* was fused to the P-mini 35S promoter to drive the GUS expression. **(B)** GUS enzymatic activity resulting from the transiently transformed tobacco leaves with constructs P-mini 35S and P-113 bp mini 35S under both normal and 200 mM NaCl or 18% (w/v) PEG 6000 treatment for 24 h. Results are mean ± standard deviation from three experiments (*n* = 15). The asterisk indicates a significant difference at *P* < 0.05.

To avoid the salinity or osmotic stress response element being at the right boundary of 110 bp (-467 to -357 bp) DNA sequence, we extended three bases to the right. To investigate if the 113 bp region (-467 to -355 bp, relative to the translational initiate site ATG) identified in the *pZmPIS* was sufficient for the salinity or osmotic stress response, we constructed a P-113 bp mini 35S vector and detected its activity under normal and salinity or osmotic stress conditions by *Agrobacterium*-mediated GUS transient assay in tobacco leaves (**Figure 7**). Under normal condition, p-113 bp mini 35S promoter had a higher activity than the P-mini 35S promoter. Bioinformatic analysis showed that the 113 bp fragment contains a CAAT-box with enhancer function, which may result in the increment of its promoter activity. GUS expression intensity of the P-113 bp mini 35S of transiently transfected tobacco leaves had a significant increment under salinity or osmotic stress condition, while the activity of the P-mini 35S promoter was not obviously changed after salt or osmotic stress treatment (**Figure 7B** and Supplementary Figure S2). Therefore, the 113 bp region (-467 to -355 bp) was sufficient for mediating salinity and osmotic stress transcriptional responses.

To better detect the transformation efficiency, the tobacco leaves infiltrated by *CaMV35S::GUS* fusion plasmid and the untransformed tobacco leaves (WT) were also used in each experiment as positive and negative controls, respectively. These leaves infiltrated with *CaMV35S* promoter plasmid had a very strong GUS activity, while these untransformed leaves had no GUS activity (Supplementary Figure S2).

## Discussion

**FIGURE 8:**
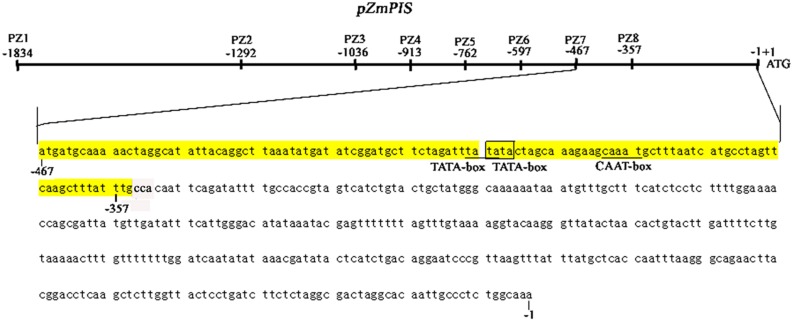
**The diagram of 467 bp (-467 to -1 bp) core promoter segment (PZ7) and 113 bp (-467 to -355 bp) key region for responding to salinity and osmotic stress of *ZmPIS***. Known *cis*-acting elements in the 113 bp region of *pZmPIS* using the PlantCARE and PLACE databases are shown in underline or border. TATA-box: core promoter element around -30 of transcription start. CAAT-box: common *cis*-acting element in promoter and enhancer regions.

Phosphoinositide signaling is involved in plant stress responses, including osmosis, temperature, drought, and salinity (König et al., 2007; Xue et al., 2007, 2009; Perera et al., 2008; Heilmann, 2009; Munnik and Vermeer, 2010). In eukaryotes, PI signaling start with PtdIns synthesis. As one of the major phospholipids, PtdIns is not only a structural component of cell membranes, but also the precursor of the several types of second messengers that allow higher plants to cope with multiple environments (Welti et al., 2002; Wang, 2004; Löfke et al., 2008). Due to the importance of PtdIns, PIS gene had brought additional attention that catalyzed the synthesis of PtdIns. Several studies showed that *PIS* participated in plant stress responses, including salinity and osmotic stress (Lin et al., 2004; Das et al., 2005; Sui et al., 2008; Zhai et al., 2012; Liu et al., 2013). In the study, we characterized its promoter to understand the functions of *ZmPIS* in response to salinity and osmotic stresses and explore its prospect for application as a stress-inducible promoter. The analysis of 5′ deleted mutants of *pZmPIS* (PZ1–PZ8) under salinity or osmotic stress conditions revealed that a 113 bp segment (-467 ∼ -355 bp, upstream of the translational initiate site ATG) is the key region for *ZmPIS* response to salinity or osmotic stress. The GUS transient assay in tobacco leaves showed that the 113 bp segment was sufficient for the response of salinity or osmotic stress. However, only two types of *cis*-acting elements including two TATA-boxes and a CAAT-box were found in the 113 bp region by online program PLACE and PLNATCARE (**Figure 8**). The TATA-box is core promoter element that functions in the transcription start of gene expression. The CAAT-box is common *cis*-acting element in promoter that general is with enhancer activity. Therefore, the 113 bp region may contain some unknown *cis*-acting elements that are crucial for *ZmPIS* response to salinity and osmotic stresses. Thus identification of the minimal *cis*-elements in this region and interacting proteins that play important roles in the regulation of *ZmPIS* promoter will be meaningful for understanding the salinity or osmotic stress responsive mechanism of plant PI signaling in future.

Promoters play important roles in initiating gene transcription and regulating gene expression temporally and spatially. Transgenic technology is an important technique for crop improvement. Application of an efficient promoter is essential to develop a vector that enables transgene expression at desired levels (Potenza et al., 2004). The development of tissue specificity or inducible promoters attracted great attention in plant genetic engineering (Kumpatla et al., 1998; Zavallo et al., 2010). The usage of inducible promoters to drive the expression of target genes under certain environment conditions can prevent unnecessary gene expression caused by constitutive promoters. In addition, transgenic safety was also seriously considered that the use of virus- or bacteria-derived promoters were limited such as *CaMV35S* (Koia et al., 2013). We identified a 467 bp (PZ7) core fragment of *ZmPIS* promoter by 5′ deleted mutant analysis. The transcription activity of PZ7 was the highest in all tissues of transgenic tobacco among PZ1–PZ8, which corresponds to about 20 and 50% of *CaMV35S* promoter under normal and salt/osmotic stress conditions, respectively. The results imply that PZ7 has high transcriptional activity, especially under salt/osmotic stress. It is known that strong expression of transgenes in plants might be also harmful for the growth and development, probably resulting in excessive waste of energy or plant morphological and physiological dysfunction (Potenza et al., 2004; Zhou et al., 2013; Han et al., 2015). In contrast with *CaMV35S*, the PZ7 might be useful for moderate transgene expression and more importantly enables the expression of transgenes at desired levels under adverse environment conditions such as salinity or osmotic stress. In addition, the size of PZ7 fragment is only 467 bp. The small plant-derived inducible promoters are useful for avoiding the repetitive usage of the constitutive promoter and reducing the vector size and thereby beneficial for genetic transformation (Han et al., 2015).

With the continuous progress of transgenic research in plants, the precise expression of exogenous genes in the host plants is increasingly required. It has been known that multiple transgenes are introduced into plants and regulated expression by the same promoter in the vector which may result in homology dependent gene silencing (Matzke and Matzke, 1995; Verdaguer et al., 1996; De Wilde et al., 2000; Han et al., 2015). Utilizing heterologous promoters to regulate the expression of target genes in transgenic receptors facilitates the prevention of gene silencing resulting from homologous promoter sequences (Kumpatla et al., 1998; Dong et al., 2015). In the study, we used heterologous promoters from maize to regulate GUS expression in tobacco and identified a 467 bp fragment (PZ7) that confers high-level and stress inducible gene expression in transgenic tobacco plants. This promoter fragment of *ZmPIS* from monocot will be useful for salt- or drought- resistance breeding in dicot crops. The transcriptional activity and expression pattern of promoters derived from different sources may also have obvious difference in dicot and monocot. In monocots, ubiquitin promoters are generally more capable of driving transgene expression than the *CaMV35S* (Christensen et al., 1992; Cornejo et al., 1993; Schledzewski and Mendel, 1994; Joung and Kamo, 2006; Kamo et al., 2012; Tao et al., 2015). Some ubiquitin promoter derived from monocots fails to direct transgene expression in dicot. The Ubi1 promoter showed less than 10% the transcriptional activity of *CaMV35S* in tobacco protoplasts (Schledzewski and Mendel, 1994). However, some promoters display strong transcriptional activity generally in both dicot and monocot. For example, a 0.3 kb *AtCTTP* promoter shows high transcriptional activity in *Arabidopsis* (dicot) and *creeping bentgrass* (monocot), implying that the small promoter can be used in both dicot and monocot plant transformation (Han et al., 2015). In the study, the PZ7 fragment of *ZmPIS* promoter derived from maize (monocot) displays strong transcriptional activity in tobacco (dicot), especially under salt or osmotic stress. Further characterization of transcriptional activity and expression patterns of PZ7 promoter in maize and evaluation of its application prospect in genetic improvement of monocot crops will also be meaningful.

In Summary, the *pZmPIS* promoter integrates multiple signal pathways that regulate plant developmental processes and allow plants to cope with multiple environments. A series of known *cis*-acting elements that enables the induced expression of *ZmPIS* by multiple abiotic stresses and hormones were present in its promoter sequence, but no salinity or osmotic stress related *cis*-regulatory element was found. In the present study, our results indicated that *pZmPIS* could well respond to NaCl or PEG stimulation by GUS histochemical staining and fluorometric assay. A 113 bp segment (-467 to -355 bp) be identified as key region for the response to salinity/osmotic stress by 5′ deleted mutation analysis of *pZmPIS* (**Figure 8**), which are useful for the future studies on *ZmPIS* interactions with other proteins involved in salt or osmotic stress signaling pathways. Furthermore, a 467 bp (-467 to -1 bp) fragment upstream from the transcription start site of *ZmPIS* could drive transgene high-level expression in an inducible manner in tobacco leaves and may be utilized as a novel salinity or osmotic stress inducible promoter system in the development of transgenic plants (**Figure 8**). Abiotic stress adaptability in crop is a complex trait, and single transgene introduction may not be sufficient to improve crop stress tolerance under natural conditions. To express multiple trangsgenes in an inducible manner will help to improve the adaptability of crops to adversity. The small PZ7 promoter could be of great use to drive transgenes expression at desired levels based on its promoter activity and inducibility.

## Author Contributions

HZ, JH, and PJ performed the isolation and functional analysis of promoter *pZmPIS*; SQ participated in data analysis; CX and QH critically revised the manuscript; ZD, ZW, and KZ participated in experimental design and manuscript proof reading; KL conceived and designed research, and drafted the manuscript. All authors read and approved the manuscript.

## Conflict of Interest Statement

The authors declare that the research was conducted in the absence of any commercial or financial relationships that could be construed as a potential conflict of interest.

## Footnotes

1 http://www.dna.affrc.go.jp/PLACE/

2 http://bioinformatics.psb.ugent.be/webtools/plantcare/html/
