# Supplementary material for: Identification of a 467 bp Promoter of Maize Phosphatidylinositol Synthase Gene (ZmPIS) Which Confers High-Level Gene Expression and Salinity or Osmotic Stress Inducibility in Transgenic Tobacco
Source: Front Plant Sci. 2016 Feb 1;7:42. doi: 10.3389/fpls.2016.00042 (PMC4740949; doi:10.3389/fpls.2016.00042)
Supplement: Supplementary file 1 [file Image_1.PDF]

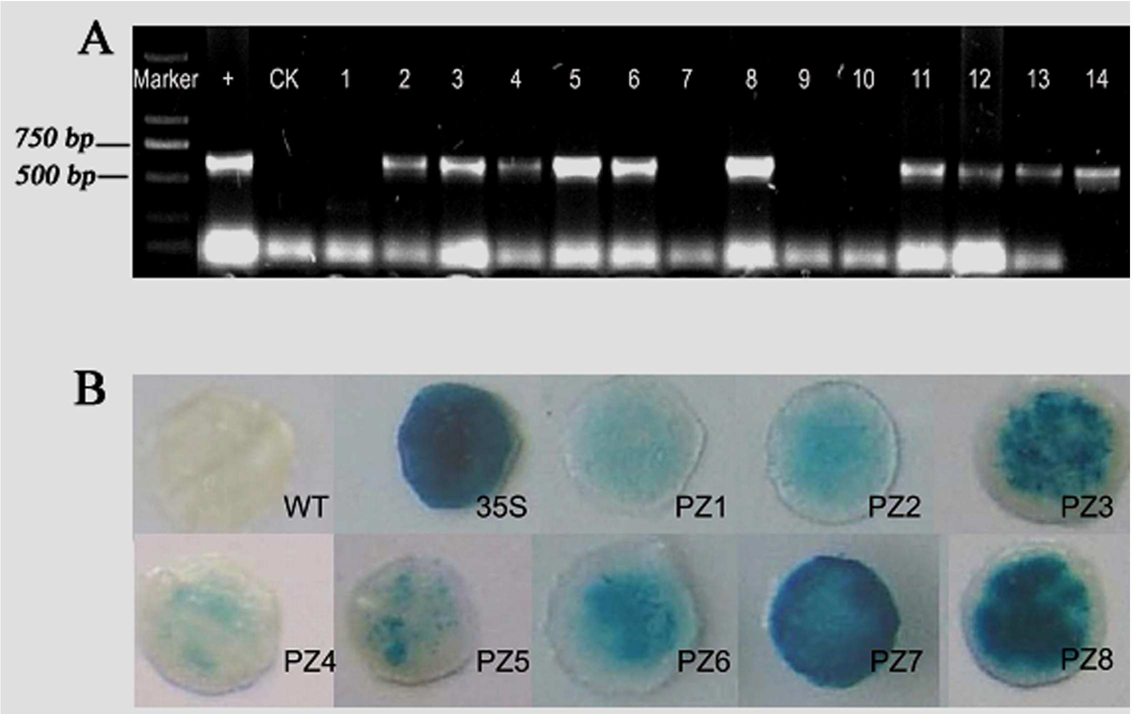

Figure S1 The PCR analysis and histochemical GUS staining of transgenic tobacco plants. **(A)** Genomic PCR analysis of transformed tobacco plants using primers HPTFR (Table 1) designed for the *hygromycin* gene. Marker, DL2000; +, the PCR result of plasmid pCAMBIA1391Z; CK, non-transformed control; 1-14, transformed T0 tobacco plants. **(B)** Histochemical GUS staining of transgenic tobacco plants. WT, non-transformed tobacco; 35S, transgenic tobacco of *CaMV35S* promoter; PZ1-PZ8, transgenic tobacco of eight truncated promoters of *pZmPIS::GUS*.

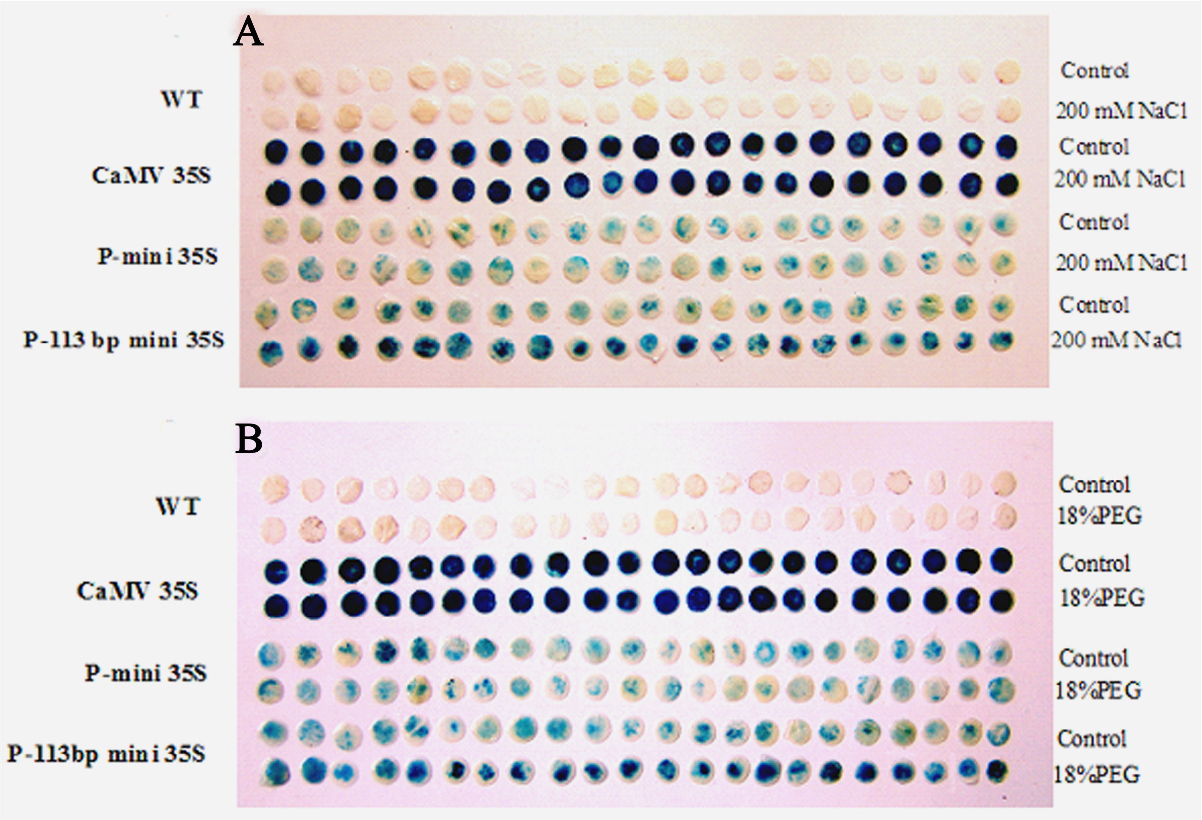

Figure S2 GUS staining of tobacco leaves in transient assays with different constructs. The *CaMV35S* represent full-length 35S promoter drive GUS expression. P-mini 35S represent the mini 35S (-46 to +10 bp) promoter drive GUS expression. The test construct P-113 bp mini 35S in which the 113 bp region (-467 to -355 bp) identified in the *pZmPIS* was fused to the P-mini 35S promoter to drive the GUS expression. **(A)** GUS histochemical staining resulting from non-transformed tobacco leaves (WT) and the transient transformed tobacco leaves with constructs *CaMV35S*, P-mini 35S and P-113 bp mini 35S under both normal and 200 mM NaCl treatment for 24 h. **(B)** GUS histochemical staining resulting from non-transformed tobacco leaves (WT) and the transient transformed tobacco leaves with constructs *CaMV35S*, P-mini 35S and P-113 bp mini 35S under both normal and 18% (w/v) PEG 6000 treatment for 24 h.
